# Intestinal Parasitic Infection in Children and Adolescents With Ocular Diseases: The Prevalence, Spectrum, and Effect of Lockdown During the COVID-19 Pandemic

**DOI:** 10.7759/cureus.60152

**Published:** 2024-05-12

**Authors:** Koushik Biswas, Biswajit Biswas, Vipin Kumar

**Affiliations:** 1 Biochemistry, All India Institute of Medical Sciences, Rae Bareli, Rae Bareli, IND; 2 Pathology, Regional Institute of Ophthalmology, Kolkata, IND; 3 Biochemistry, Uttar Pradesh University of Medical Sciences, Saifai, IND

**Keywords:** india, covid-19 lockdown, seasonal trend, adolescents, soil transmitted helminths, covid-19 pandemic, ocular disease, children, intestinal parasites, prevalence

## Abstract

Introduction: People with visual impairments and blindness face challenges in performing regular tasks such as maintaining proper sanitation, which makes them vulnerable to intestinal parasitic infections.

Aims and objectives: This study aims to examine the prevalence and distribution of intestinal parasitic infections in children and adolescents with ocular diseases and to assess if the lockdown during the COVID-19 pandemic affected these rates.

Methods: This retrospective, hospital record-based study was conducted among children and adolescents attending the Regional Institute of Ophthalmology in Kolkata, India. It involved routine stool examinations as part of their treatment during 2019-2020. Early morning stool specimens were collected and brought to the institute laboratory in containers. Stools were examined under a microscope for cysts, ova, parasites, and adult worms. Findings were recorded in the laboratory record book. These data were then extracted into a spreadsheet and analyzed using IBM SPSS Statistics for Windows, Version 26 (Released 2019; IBM Corp., Armonk, New York).

Results: The prevalence of intestinal parasitic infections was 8.59% (59 out of 687 patients). Among those 59 positive cases, *Ascaris lumbricoides*, *Giardia lamblia*, *Entamoeba histolytica*, *Trichuris trichiura*, *Taenia *spp., *Enterobius vermicularis*, and *Isospora belli *were detected in 27 (45.8%), 15 (25.4%), 8 (13.6%), 6 (10.2%), 3 (5.1%), 2 (3.4%), and 1 (1.7%) patients, respectively. The positivity rate of stool samples was higher from September and thereafter from January to March. The sample positivity rate was higher post-pandemic and lockdown, but not statistically significant (11.5% vs. 5.3%; χ²=4.044, df=1, p=0.44).

Conclusion: *Ascaris lumbricoides* was the most commonly observed intestinal parasite in children and adolescents with ocular disease in our setting. Seasonal variation was noted with higher case positivity at the end of the rainy season and thereafter in winter. Therefore, we propose to strengthen the routine deworming program during this period in Eastern India. Higher sample positivity after the pandemic may be attributed to school closures during the lockdown period, which might have caused some children to miss their routine deworming medication.

## Introduction

Intestinal parasites affect approximately 3.5 billion people globally, leading to illness in about 450 million people annually. Children constitute the majority of those affected by these parasites [1]. The disease is prevalent in tropical and subtropical areas, with a significant number of cases in underdeveloped countries [2]. Around a quarter of the global cases of soil-transmitted helminth (STH) infections are reported from South Asia, with India experiencing the highest number in this region [3]. According to 2023 reports, 336.1 million children in India require preventive chemotherapy annually [4]. The most common intestinal parasites include roundworm (*Ascaris lumbricoides*), whipworm (*Trichuris trichura*), and hookworm (*Ancyclostoma duodenale* and *Necator americanus*) [5]. Their transmission follows a specific sequence: (i) open defecation by infected individuals leads to the excretion of worm eggs into the soil; (ii) optimum temperature and moisture facilitate the maturation of these eggs into infectious worm eggs (roundworm and whipworm) or larvae (hookworms); and (iii) infection occurs when humans ingest these eggs through contaminated vegetables/fruits, drinking water, or through skin penetration by larvae residing in the soil [6].

Once inside the body, these worms feed on host tissues and blood, leading to iron and protein loss [7]. Diarrhea, abdominal pain, and anemia are common manifestations of the infection. However, long-term infections in children can lead to reduced cognitive abilities, intellectual capacity, and work productivity [8]. The prevalence of STH infections is influenced by factors such as sanitation, hygiene practices, water quality, and socioeconomic status [9]. The 2019-2020 Demographic and Health Survey (DHS) indicated that 25.4% of households in rural India practiced open defecation [10]. The warm and moist tropical climate of India provides an ideal environment for the survival and maturation of STH eggs or larvae [11]. Under the National Iron Plus Initiative for Anemia Control program, biannual deworming with albendazole is mandated for individuals aged one to 19 years. School-going children receive this deworming medication through government-run schools [12].

As of 2021, 1.24% of children aged 0-6 years in India are disabled, with 30% of them having a visual disability [13]. People with disabilities face challenges in maintaining proper sanitation, making them susceptible to intestinal parasitic infections [14]. While the prevalence of intestinal parasitic infection in children with mental disabilities has been studied [15], there is a lack of knowledge regarding its prevalence among children and adolescents with ocular diseases, which places them at risk of ocular disability. In this study, we aim to study the prevalence and distribution of intestinal parasitic infections in children and adolescents with ocular diseases and to assess if the lockdown during the COVID-19 pandemic affected it.

## Materials and methods

Study setting

This study was conducted at the Regional Institute of Ophthalmology, Kolkata, the largest government-run tertiary care ophthalmology hospital in Eastern India, which has a 300-bed capacity. Approximately 800 patients visit the outpatient department (OPD) daily, and the institute also operates a specialized clinic for pediatric ophthalmology. The study took place in the central laboratory of the institute, equipped to carry out routine investigations for ophthalmology patients, from January 2019 to December 2020.

Study design

This is a retrospective, hospital record-based study. The inclusion criteria were children and adolescents (<18 years of age) who attended the institute with ocular diseases and subsequently underwent a routine stool examination at the institute laboratory as part of their pre-anesthetic work-up before ocular surgery. We included findings from stool tests conducted from January 1, 2019, to December 31, 2020. There were no exclusion criteria.

Study duration

The retrospective data were collected from January 2019 to December 2020. The data analysis was conducted during the year 2021.

Sampling

The prevalence of intestinal parasitic infections among children and adolescents with ocular disease is unknown. Assuming a general prevalence of intestinal parasitic infections in children at 17% based on a previous study [16], with a 95% confidence level and a 5% margin of error, we calculated the required sample size to be 217.

Study procedure

A fresh stool sample (approximately 20 grams) was collected early in the morning and brought by the child’s parent or guardian in a container to the central laboratory. A drop of saline was placed on the left half of a microscope slide, and one drop of iodine on the right half. A small portion of the stool sample, equivalent to the size of a match head, was picked up with an applicator stick and mixed with a drop of saline. Another small portion was similarly mixed with the drop of iodine. Two coverslips were then placed on each half. The samples were examined under a microscope by trained laboratory personnel, identifying cysts, ova, trophozoites, and adult worms based on their characteristic features [17].

Data extraction

Data from all patients undergoing the stool examination, including their age and sex, findings from the microscopic stool test, and the date of the test, were retrieved from laboratory records and extracted into a Microsoft Excel sheet (Microsoft Corporation, Redmond, Washington).

Ethical considerations

Ethical clearance was obtained from the Institutional Ethical Committee (Memo No. RIO/IEC/ACD/2021/29) before commencing the study. The study adhered to the guidelines of the Declaration of Helsinki.

Statistical analysis

Strict confidentiality was maintained regarding patient data utilized in the study. Prevalence was calculated by dividing the number of patients with a positive stool test for intestinal parasites by the total number of patients tested. All categorical data were presented as frequency and percentage. Pearson’s chi-square test was employed to identify any statistically significant differences between the categorical data. A p-value of <0.05 was considered statistically significant. The sample positivity rate for a particular month or period was determined by dividing the number of positive tests by the total number of tests conducted during that timeframe. Seasonal trends were analyzed by comparing monthly sample positivity rates for intestinal parasites. The sample positivity rate of intestinal parasitic infections during the pre-pandemic period (June-December 2019) was compared with the post-pandemic and post-lockdown period (June-December 2020) to assess the impact of the COVID-19 pandemic and lockdown. Data were analyzed using IBM SPSS Statistics for Windows, Version 26 (Released 2017; IBM Corp., Armonk, New York).

## Results

In this study, we analyzed data from 687 children and adolescents (<18 years old) with ocular diseases who underwent stool examinations from January 2019 to December 2020. These records were retrieved from laboratory archives, tabulated, and analyzed. No duplicate or repeat stool test reports were observed among the patients. Out of 687 patients, 59 tested positive for intestinal parasites, yielding a prevalence of 8.59% ((59/687) x 100 = 8.59%). Upon age-wise analysis, the prevalence of intestinal parasitic infection was 6.35% in the <5 years age group, 11.33% in the 5-10 years age group, and 8.51% in the 10-18 years age group. Although the prevalence was highest in the 5-10 years age group, the difference was not statistically significant. Sex-wise analysis revealed that the prevalence of intestinal parasitic infection was 8.89% in male patients and 8.12% in female patients. No significant statistical difference was found in the prevalence of infection between the two sexes (Table 1).

**Table 1:** Age- and gender-wise prevalence of intestinal parasitic infection in children and adolescents with ocular diseases

		Prevalence	Pearson’s chi-square	df	p-value
Age group	<5 years	19/299 (6.35%)	4.277	2	0.118
	5-10 years	28/247 (11.33%)			
	10-18 years	12/141 (8.51%)			
	Total	59/687 (8.59%)			
Sex	Male	37/416 (8.89%)	0.126	1	0.732
	Female	22/271 (8.12%)			
	Total	59/687 (8.59%)			

Upon further analysis of the 59 patients with positive stool tests, seven different types of intestinal parasites were identified. *Ascaris lumbricoides* was the most predominant intestinal parasite, found in 27 out of 59 patients (45.8%). It was followed by *Giardia lamblia* in 15 patients (25.4%), *Entamoeba histolytica *in 8 (13.6%), *Trichuris trichiura *in 6 (10.2%), *Taenia *spp. in 3 (5.1%), *Enterobius vermicularis *in 2 (3.4%), and *Isospora belli *in 1 patient (1.7%) (see Table 2). Among these 59 cases, 56 patients were infected with a single type of intestinal parasite, while three patients had infections with two different intestinal parasites.

**Table 2:** Distribution of intestinal parasites in children and adolescents whose stool test was positive (n=59)

Intestinal parasite	n (%)
Ascaris lumbricoides	27 (45.8)
Giardia lamblia	15 (25.4)
Entamoeba histolytica	8 (13.6)
Trichuris trichura	6 (10.2)
Taenia	3 (5.1)
Enterobius vemicularis	2 (3.4)
Isospora belli	1 (1.7)

On analyzing the seasonal trend, we observed that the positivity rate of intestinal parasites in stool samples tested was higher in September and thereafter from January to March (Table 3).

**Table 3:** Seasonal trend of intestinal parasite positivity rate in stool sample Positivity rate = (p/n) × 100%

Month	Sample positive (p)	Sample tested (n)	Positivity rate
January	13	106	12.3
February	9	81	11.1
March	9	80	11.3
April	3	49	6.1
May	0	13	0
June	4	36	11.1
July	0	63	0
August	1	61	1.6
September	9	60	15.0
October	0	33	0
November	7	56	12.5
December	4	49	8.2
Total	59	687	8.6

There was a nationwide lockdown across India from March 25, 2020, to May 31, 2020, due to the COVID-19 pandemic. We compared the prevalence of intestinal parasitic infections during the pre-pandemic period (June-December 2019) with the post-pandemic and post-lockdown period (June-December 2020). The results showed that the prevalence of intestinal parasitic infections was higher after the pandemic and lockdown than during the pre-pandemic period (11.5% vs. 5.3%). However, this increase was not statistically significant (χ² = 4.044, df = 1, p = 0.44) (see Table 4).

**Table 4:** Comparison of intestinal parasitic infection among patients with ocular disease before and after COVID-19 pandemic Positivity rate = (p/n) × 100%

	Pre-pandemic (June-December 2019)	Post-pandemic (June-December 2020)	Pearson chi-square value	df	p-value
Sample positive (p)	14	11	4.044	1	0.44
Sample tested (n)	262	96			
Positivity rate	5.3	11.5			

## Discussion

Many children and adolescents with ocular diseases face various degrees of visual impairment, making it difficult for them to maintain proper hygiene, which is crucial for preventing intestinal parasitic infections. To the best of our knowledge, there has been no study to date on the prevalence of intestinal parasitic infections in children and adolescents with ocular diseases. These individuals frequently attend our institute for consultation as it is a tertiary care ophthalmology center. The clinical diagnosis ranges from benign conditions like cataracts, chalazion, and squint to malignant conditions like retinoblastoma. All such patients, who required surgical intervention, were advised to undergo a routine stool examination as part of the pre-anesthetic checkup to prevent potential airway obstruction by helminths during anesthesia, which could pose intraoperative risks [18]. In this study, we observed that the prevalence of intestinal parasitic infection in children and adolescents with ocular diseases was 8.59%. Age-wise, the prevalence was 6.35% in children under five years of age (Table 1). Banerjee et al. [16] reported that the prevalence of intestinal parasitic infection in under-five children was 17%. This may be because children with ocular diseases, especially those with visual impairments, are less likely to play outdoors, which may indirectly reduce their exposure to infective parasite eggs and larvae.

In our study, the prevalence of intestinal parasitic infection was 11.33% in the 5-10 years age group. Mondal et al. [19] reported that the prevalence in 5-12-year-old school-going children was 22%. Chatterjee et al. [20] reported that the prevalence in primary school children was 27.5%. This highlights that the prevalence of intestinal parasitic infection in school-going children with ocular disease is lower than that in children of the same age group who do not have an ocular problem. This may be because ocular diseases limit children’s ability to play outdoor games [21], which in turn reduces their exposure to infective parasite eggs and larvae.

A study conducted on blind people in Eastern Nepal reported that 31.1% of adult blind people had intestinal parasitic infections [14]. Compared to this, the prevalence was lower in our study. One reason for this may be that our study included patients younger than 18 years of age who received biannual deworming under the government program. Furthermore, the Swachh Bharat Abhiyan program [22] launched by the Government of India in 2014, which provides funds for the construction of toilets in villages and prevents open defecation, might have helped to reduce the prevalence of intestinal parasitic infection in India, compared to neighboring countries. We did not observe any significant statistical difference in the prevalence of intestinal parasites in stool between the two sexes (Table 1). Similar observations were reported in previous studies as well [16,23,24].

The most frequent intestinal parasite observed in our study was *Ascaris lumbricoides*, followed by *Giardia lamblia* and *Entamoeba histolytica*. Banerjee et al. [16] reported *Giardia lamblia* as the most frequent parasite. Mondal et al. [19] reported *Ascaris lumbricoides* as the most frequent parasite. Chatterjee et al. [20] reported *Giardia intestinalis* as the most frequent parasite. A comparison of these studies is presented in Table 5.

**Table 5:** Comparison of intestinal parasite distribution in different studies

	Current study	Banerjee et al. [16]	Mondal et al. [19]	Chatterjee et al. [20]
Study group	Hospital-based	Community-based	Hospital-based	Community-based
Study design	Cross-sectional retrospective	Cross-sectional prospective	Cross-sectional prospective	Cross-sectional prospective
Duration	January 2019 to December 2020	November 2018 to April 2019	July 2016 to December 2016	November 2013 to July 2014
Place	Kolkata (West Bengal)	Purba Bardhaman (West Bengal)	Nadia (West Bengal)	Midnapore (West Bengal)
Sample size	687	294	103	560
Prevalence	8.58% (59 out of 687)	17% (50 out of 294)	22.33% (23 out of 103)	27.5% (154 out of 560)
Ascaris lumbricoides	45.8%	6%	33.3%	12.3%
Giardia lamblia	25.4%	48%	11.1%	50%
Entamoeba histolytica	13.6%	36%	18.5%	12.3%
Trichuris trichura	10.2%	10%	11.1%	5.9%

The majority of the patients attending our institution are from the state of West Bengal, located in the eastern part of India. The Gangetic West Bengal region has four distinct seasons. The winter season, from December to February, is followed by a pre-monsoon season (hot weather season) from March to May. Thereafter, the period from June to mid-September constitutes the southwest monsoon (rainy season) and the period from the latter half of September to November is the post-monsoon period [25]. In our study, we observed a seasonal trend in the positivity rate of intestinal parasites in stool samples, with a higher positivity rate in September and thereafter from January to March (Table 3). It is reported that temperatures above 37°C prevent the hatchability of gastrointestinal helminths [26]. Hence, the extreme temperatures (39-45°C) during summer and early monsoon create an unfavorable environment for the hatching of helminth eggs, thereby contributing to the lower positivity rate of intestinal parasites in the summer and early monsoon (April-August).

In our study, the intestinal parasite positivity rate in stool was significantly higher after the COVID-19 pandemic and lockdown compared to the pre-pandemic period (11.5% vs. 5.3%; χ^2^ = 4.044, df=1, p = 0.44). Two previous studies have also reported a surge in intestinal parasitic infections with the waning of the epidemic [27, 28]. This may be because the existing manpower in the health sector in India was utilized to tackle the COVID-19 pandemic, and there was less focus on tackling intestinal parasitic diseases. Also, as schools were temporarily shut down during the first wave of the pandemic in India (March 2020 to June 2020), school-going children might have missed the deworming dose of albendazole, which is provided to them by their schools.

Our study was not without limitations. As it was a retrospective laboratory record-based study, we could not include the clinical findings of the patient. Our institution lacked an online patient management system, from which clinical data of past patients could be retrieved. We did not have facilities for modified acid-fast staining, which prevented us from determining Coccidian parasites in stool. Molecular biology methods to determine intestinal parasites in stool specimens were not available at our institution.

## Conclusions

To the best of our knowledge, this is the first study reporting the prevalence and distribution of intestinal parasitic infection in children with ocular diseases. We further tried to analyze the seasonal pattern of intestinal parasitic infection and the impact of the COVID-19 pandemic and lockdown. In our setting, we observed that Ascaris lumbricoides was the most common infection in children and adolescents with ocular diseases. The lower prevalence of intestinal parasitic infection in children and adolescents with ocular diseases may be related to their restricted outdoor mobility due to visual impairment of varying extents. Based on the seasonal trend, we suggest that providing deworming medication (albendazole) during September (post-monsoon) and again in January or February (winter) may be more effective in preventing intestinal parasitic infection among children in the state of West Bengal, located in eastern India. Although statistically insignificant, the surge in intestinal parasitic infection after the COVID-19 pandemic deserves special attention and needs to be studied further. The health department should promote the existing deworming program at the village level and in government-run schools to reduce the prevalence of intestinal parasitic infection. Due to a lack of clinical data, we could not relate the clinical symptoms of patients with their laboratory reports.
